# Co-aggregation and heritability of organ-specific autoimmunity: a population-based twin study

**DOI:** 10.1530/EJE-20-0049

**Published:** 2020-03-04

**Authors:** Jakob Skov, Daniel Eriksson, Ralf Kuja-Halkola, Jonas Höijer, Soffia Gudbjörnsdottir, Ann-Marie Svensson, Patrik K E Magnusson, Jonas F Ludvigsson, Olle Kämpe, Sophie Bensing

**Affiliations:** 1 Department of Molecular Medicine and Surgery, Karolinska Institutet, Stockholm, Sweden; 2 Department of Medicine, Karlstad Central Hospital, Karlstad, Sweden; 3 Center for Molecular Medicine, Department of Medicine (Solna), Karolinska Institutet, Stockholm, Sweden; 4 Department of Endocrinology, Inflammation and Infection Theme, Karolinska University Hospital, Stockholm, Sweden; 5 Department of Medical Epidemiology and Biostatistics, Karolinska Institutet, Stockholm, Sweden; 6 Department of Surgical Sciences, Uppsala University, Uppsala, Sweden; 7 Departent of Molecular & Clinical Medicine, Institute of Medicine, University of Gothenburg, Gothenburg, Sweden; 8 Swedish National Diabetes Register, Västra Götalandsregionen, Gothenburg, Sweden; 9 Department of pediatrics, Örebro University Hospital, Örebro, Sweden; 10 K.G. Jebsen Center for Autoimmune Diseases, University of Bergen, Bergen, Norway

## Abstract

**Objective:**

Co-aggregation of autoimmune diseases is common, suggesting partly shared etiologies. Genetic factors are believed to be important, but objective measures of environmental vs heritable influences on co-aggregation are absent. With a novel approach to twin studies, we aimed at estimating heritability and genetic overlap in seven organ-specific autoimmune diseases.

**Design:**

Prospective twin cohort study.

**Methods:**

We used a cohort of 110 814 twins to examine co-aggregation and heritability of Hashimoto’s thyroiditis, atrophic gastritis, celiac disease, Graves’ disease, type 1 diabetes, vitiligo and Addison’s disease. Hazard ratios (HR) were calculated for twins developing the same or different disease as compared to their co-twin. The differences between monozygotic and dizygotic twin pairs were used to estimate the genetic influence on co-aggregation. Heritability for individual disorders was calculated using structural equational modeling adjusting for censoring and truncation of data.

**Results:**

Co-aggregation was more pronounced in monozygotic twins (median HR: 3.2, range: 2.2–9.2) than in dizygotic twins (median HR: 2.4, range: 1.1–10.0). Heritability was moderate for atrophic gastritis (0.38, 95% CI: 0.23–0.53) but high for all other diseases, ranging from 0.60 (95% CI: 0.49–0.71) for Graves’ disease to 0.97 (95% CI: 0.91–1.00) for Addison’s disease.

**Conclusions:**

Overall, co-aggregation was more pronounced in monozygotic than in dizygotic twins, suggesting that disease overlap is largely attributable to genetic factors. Co-aggregation was common, and twins faced up to a ten-fold risk of developing diseases not present in their co-twin. Our results validate and refine previous heritability estimates based on smaller twin cohorts.

## Introduction

Twin studies have been essential in charting the relative importance of environmental and genetic factors in disorders with complex inheritance (1). Despite the high heritability demonstrated in most autoimmune diseases (AIDs), twin pairs are often discordant – that is, even if one twin develops an autoimmune disease, the co-twin will generally not acquire this particular disease (2). This discordance reflects the importance of non-genetic factors. However, the absence of one specific autoimmune disorder in a co-twin does not necessarily equate to absence of *any* autoimmune disease.

Clustering of different AIDs in individuals and families is common, underlining their partially shared etiology (3, 4), but the relative influence of genetic and environmental factors on co-aggregation is still largely unknown. Genetic studies provide ample evidence of pleiotropy in autoimmunity (5, 6), but many risk alleles with opposite effect in different disorders have also been identified, conferring increased risk of one disease but decreased risk for another. Consequently, most of the genetic predisposition to autoimmunity may be disease specific rather than pleiotropic (5, 6, 7). Epidemiological data also point to the importance of non-genetic effects in disease clustering, with stronger associations observed between siblings (that share environment to a greater extent) than parent–offspring pairs (3). Given the complex overlap of AIDs, epidemiological studies specifically designed to illuminate risk factors shared between diseases are warranted. In twin studies, the difference between monozygotic (MZ) and dizygotic (DZ) pairs can further partition shared components into genetic and environmental, respectively. Moreover, studying AIDs in a wider context can provide us with risk estimates that are valuable to patients and physicians – the risk of acquiring not only one specific disease, but rather the risk of acquiring any one in a cluster of related disorders. Despite this, twin studies in autoimmunity have focused on quantifying the genetic influence in merely a single disease at the time. To the best of our knowledge, no attempts of cross-twin-cross-trait analyses have so far been undertaken.

In this study, we aimed at exploring the influence of genetic and environmental components on a set of related AIDs, both within and across diseases. By using a large twin cohort, we could in parallel estimate heritability and cross-trait aggregation in seven organ-specific autoimmune diseases, namely Hashimoto’s thyroiditis (HT), Graves’ disease (GD), type 1 diabetes (DM), vitiligo (VI), Addison’s disease (AD), atrophic gastritis/pernicious anemia, and celiac disease (CD), all known to cluster to various degrees (8, 9, 10, 11, 12, 13). With state-of-the-art twin statistics, we sought to confirm and refine heritability estimates for previously investigated diseases (14, 15, 16, 17, 18) and to provide first time data on heritability for diseases yet unexplored.

## Subjects and methods

### Study population

The Swedish Twin Registry (STR) contains data on more than 85 000 twin pairs, with zygosity determined using a validated intra-pair similarity algorithm, DNA, or opposite sex (19). We identified all twins from complete twin pairs, born in Sweden between 1886 and 2006. To improve diagnostic precision and ensure coverage of national health registries, both twins were required to be alive or not yet born in 1976. We used information on both same-sex and opposite-sex twin pairs in all analyses. The primary reason for this was to optimize power, as some of the diseases examined are rare. To distinguish DM from type 2 diabetes and other forms of non-insulin-dependent diabetes, we cross-matched the STR with the National Diabetes Register (NDR) (20). For HT, GD, CD, VI, atrophic gastritis, and pernicious anemia, diagnoses were ascertained through recordings of relevant ICD-codes in the National Patient Register (NPR) from 1964 to 2015 (21). Atrophic gastritis and pernicious anemia are ICD-coded separately but were considered as the same autoimmune disease, hereafter referred to as AG. ICD-codes indicating non-autoimmune etiology were used as exclusions to improve diagnostic accuracy. If patients had multiple (≥2) ICD-codes representing the same disease, multiple (≥2) exclusion codes were required for exclusion. The Prescribed Drug Register (PDR), started in 2005, was used for additional diagnostic precision (22). Multiple (≥2) dispensations of levothyroxine (Anatomical Therapeutic Chemical code (ATC) H03AA) were required for a diagnosis of HT and multiple (≥2) prescriptions of vitamin B12 (ATC B03BA) for a diagnosis of AG in patients alive after December 2005. Ascertainment of autoimmune AD followed detailed criteria stipulated in a previous work, with minor adjustments (18). An aggregate of all seven diseases, referred to as ‘any disease’, was constructed by combining inclusion criteria for individual AIDs. Complete inclusion and exclusion criteria for all diagnoses are listed in the Supplementary material (see section on supplementary materials given at the end of this article). For each disease we identified the first date of observed diagnosis or prescription to be used as disease onset in statistical analyses.

### Descriptive statistics

Data for the twin cohort was summarized according to autoimmune status (prevalent/non-prevalent) and further categorized by sex and zygosity. Prevalence estimates (observed diagnosis during follow-up), expressed as cases per 100 000 individuals, were calculated for each individual disorder and for ‘any disease’. Results were separated per sex. For each disorder, including ‘any disease’, we subsequently calculated number of twin pairs concordant unaffected, discordant, and concordant affected subdivided by zygosity. We then calculated the probandwise concordance rate, that is, the proportion of twins who had a disorder if their co-twin had the same disorder. The probandwise concordance rate may be contrasted to the population prevalence, to evaluate the excess risk for a disorder when one’s co-twin is affected. AIDs were considered as binary traits (affected or not affected), therefore, the liability-threshold approach was used in analyzing the data. This method assumes an underlying normal distribution of the liability of disease and that individuals with a liability beyond a certain threshold develop the disorder. The inferred correlation between liabilities in co-twins is equivalent to a tetrachoric correlation. This correlation is the basis of subsequent heritability analysis.

### Familial co-aggregation

Using date of birth, year of start of observation (separate years per different diagnoses), date of diagnosis, or date of right-censoring, we calculated each individual’s age at risk and age at outcome or censoring. In Cox regression, we used this information to estimate the hazard ratio (HR) of one twin having the outcome, while the co-twin’s disease status was used as exposure. Each twin was considered unexposed during the time the co-twin had not had the disorder and exposed after her/his date of diagnosis. We accounted for left truncation by allowing different ages of entry into analyses. The HR is thus the increased risk of diagnosis after one’s co-twin has had a diagnosis. We performed this analysis separately for MZ and DZ twin pairs and adjusted for sex and birth year categories. The outcomes of developing the same disease as found in the co-twin, a different disease than found in the co-twin, and any of the studied diseases were analyzed. HRs for developing a different disorder and any disorder were used as measures of co-aggregation. All aggregation analyses were performed with package ‘survival’ in the software R (23, 24, 25), accounting for dependency between twins in pairs using a cluster-robust sandwich estimator.

### Heritability

The liability-threshold model, accounting for left truncation and right censoring, was used for calculating heritability (26). We fitted ACE models, that is, models with variance contributions from additive genetic (A), shared environment (C), and non-shared environment (E), ADE models (where D is dominance genetic deviations), and AE models, for individual AIDs, as well as for ‘any disease’. We compared the fitted models using Akaike’s Information Criterion (AIC) and presented the best-fitting model for each outcome. All analyses were adjusted for sex and birth year categories (complete adjusted and unadjusted models, with and without left truncation and right-censoring, are found in Supplementary Tables 1a, b, c and d). These analyses were performed using the ‘mets’ library in the software R (25, 26, 27). Attempts of using bivariate analysis to estimate shared genetic and environmental components across disease pairs were mostly unsuccessful due to insufficient data (too few twin pairs with a different AID in the co-twin).

The study was approved by the ethics committee in Stockholm, Sweden. Informed consent was waived by the ethics committee.

## Results

A total of 120 286 individuals that were part of complete twin pairs were identified in the STR. Of these, 3966 individuals were excluded due to unknown zygosity. A further 3453 individuals deceased before 1976 were excluded along with the 2049 co-twins of deceased individuals. Two pairs (four individuals) with ambiguous birth data were also excluded, resulting in a final sample of 110 814 twins.

### Descriptive statistics

In total, 1322 autoimmune diagnoses were recorded among 35 990 MZ twins and 2783 diagnoses were recorded among 74 820 DZ twins. Sex and zygosity among individuals affected and unaffected by autoimmunity are presented in Table 1. Prevalence estimates for individual AIDs and ‘any disease’ are listed in Table 2. HT, AG, CD, GD, and ‘any disease’ were all more common in women than in men, whereas no statistically significant sex differences were observed for DM, VI, or AD. In all, 291 twin pairs were concordant (same AID in co-twin) for disease and 153 twin pairs displayed cross-trait concordance (different AID in co-twin). All disease combinations are presented in Supplementary Fig. 1. Probandwise concordance rates were higher for MZ-twin pairs than DZ-twin pairs for all disorders, as were tetrachoric correlations, indicating that genetic effects are important in all the studied AIDs (Table 3). Vitiligo and AD had the lowest prevalence and no concordant DZ-pairs.

**Table 1:** Sex and zygosity of twins. Data are presented as *n* (%).

	All	No autoimmune disease*	Autoimmune disease*
All	110 814 (100.0)	106 932 (96.5)	3882 (3.5)
Male	52 171 (47.1)	51 116 (47.8)	1055 (27.2)
Female	58 643 (52.9)	55 816 (52.2)	2827 (72.8)
Monozygotic	35 990 (32.5)	34 745 (32.5)	1245 (32.1)
Dizygotic	74 824 (67.5)	72 187 (67.5)	2637 (67.9)

*Of the seven autoimmune diseases included in the study: Hashimoto’s thyroiditis, atrophic gastritis, celiac disease, Graves’ disease, type 1 diabetes, vitiligo, and Addison’s disease.

**Table 2:** Prevalence estimates and sex distribution of autoimmune diseases.

Disease	Cases, *n* (%)	Prevalence *n*/100 000	*P*-value for sex difference
Hashimoto’s thyroiditis			<0.0001
Female	1410 (84)	2404	
Male	273 (16)	523	
Atrophic gastritis			<0.0001
Female	398 (61)	679	
Male	254 (39)	487	
Celiac disease			<0.0001
Female	433 (67)	738	
Male	215 (33)	412	
Graves’ disease			<0.0001
Female	463 (83)	790	
Male	95 (17)	182	
Type 1 diabetes			0.18
Female	179 (49)	305	
Male	185 (51)	355	
Vitiligo			0.56
Female	95 (55)	162	
Male	77 (45)	148	
Addison’s disease			0.95
Female	15 (54)	26	
Male	13 (46)	25	
Any of the above diseases			<0.0001
Female	2827 (73)	4821	
Male	1055 (27)	2022	

*P*-values were calculated using a generalized estimating equation approach with cluster-robust standard errors and logit link.

**Table 3:** Concordance, discordance, probandwise concordance rates, and tetrachoric correlations.

Disease/zygosity	Concordant unaffected pairs, *n*	Discordant pairs, *n*	Concordant affected pairs, *n*	Probandwise concordance rate	Tetrachoric correlation
Hashimoto’s thyroiditis					
MZ	17 523	391	81	0.29 (0.25–0.35)	0.70 (0.65–0.75)
DZ	36 339	1016	57	0.10 (0.08–0.13)	0.39 (0.33–0.45)
Atrophic gastritis					
MZ	17 803	183	9	0.09 (0.05–0.17)	0.47 (0.33–0.60)
DZ	36 968	437	7	0.03 (0.02–0.06)	0.25 (0.12–0.38)
Celiac disease					
MZ	17 855	93	47	0.50 (0.42–0.60)	0.90 (0.86–0.94)
DZ	36 981	401	30	0.13 (0.09–0.18)	0.55 (0.47–0.62)
Graves’ disease					
MZ	17 831	145	19	0.21 (0.14–0.30)	0.68 (0.58–0.77)
DZ	37 040	369	3	0.02 (0.01–0.05)	0.16 (0.00–0.33)
Type 1 diabetes					
MZ	17 900	74	21	0.36 (0.27–0.49)	0.83 (0.76–0.90)
DZ	37 170	236	6	0.05 (0.02–0.11)	0.39 (0.25–0.53)
Vitiligo					
MZ	17 933	56	6	0.18 (0.09–0.35)	0.69 (0.55–0.84)
DZ	37 308	104	0	NA	NA
Addison’s disease					
MZ	17 986	4	5	0.71 (0.49–1.00)	0.98 (0.94–1.00)
DZ	37 398	14	0	NA	NA
Any of the above diseases					
MZ	16 957	831	207	0.33 (0.30–0.37)	0.67 (0.63–0.71)
DZ	34 926	2335	151	0.12 (0.10–0.13)	0.30 (0.25–0.34)

NA, not applicable.

### Familial aggregation and co-aggregation

**Figure 1:**
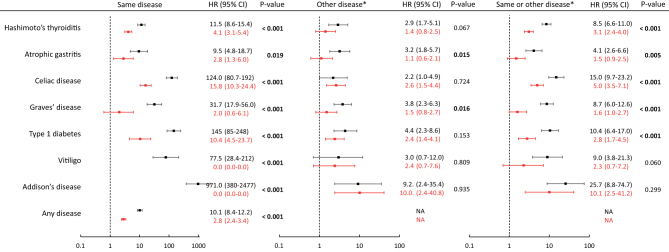
Familial aggregation and co-aggregation of autoimmunity. Hazard ratios for developing the same disease as present in the co-twin, a different disease, or any of the studied diseases. Monozygotic twins in black and dizygotic twins in red. *Any of the studied diseases.

The aggregation analyses showed increased hazard in co-twins of affected twins, with higher HRs in MZ than in DZ twins (Fig. 1) Some HRs in MZ were very high, for example, for AD (HR: 9716; 95% CI: 380–2477), reflecting a very low baseline rate and a high twin pair concordance, and prevalence was merely 0.025% (Table 2), whereas the probandwise concordance was 0.71, indicating a ‘prevalence’ of 71.4% in MZ twins whose co-twin also had AD. Co-aggregation was prevalent in all disorders, with higher HRs in MZ than in DZ twins for most, but not all, disorders (Fig. 1).

### Heritability

The AICs indicated that the AE models provided the best fit for all disorders, as well as for all combined AIDs, except for GD. However, for GD, the broad sense heritability (A+D) was similar for AE model and ADE model, therefore we present results from the AE models in Table 4 (all models are presented in Supplementary Tables 1a, b, c and d).

**Table 4:** Estimated heritability and environmental factors from best-fitting models.

Disease	Proportions of variance			
	A	95% CI	E	95% CI
Hashimoto’s thyroiditis	0.64	0.58–0.70	0.36	0.30–0.43
Atrophic gastritis	0.38	0.23–0.53	0.62	0.47–0.77
Celiac disease	0.91	0.87–0.94	0.09	0.06–0.13
Graves’ disease	0.60	0.49–0.71	0.40	0.29–0.51
Type 1 diabetes	0.81	0.73–0.89	0.20	0.12–0.27
Vitiligo	0.65	0.50–0.81	0.35	0.19–0.50
Addison’s disease	0.97	0.91–1.00	0.03	0.00–0.09
Any of the above diseases	0.69	0.65–0.73	0.31	0.27–0.35

A, additive genetic effects; E, effects from unique environment, not shared by twins. Full models are presented in Supplementary Tables 1a, b, c, and d.

## Discussion

In this study, we examined co-aggregation and heritability in seven autoimmune diseases using a large population-based twin cohort and refined statistical methods. Co-aggregation was common and more pronounced in MZ than in DZ twins, indicating that disease clustering is largely caused by genetic overlap. Moreover, our results confirm and refine previous heritability estimates. For VI and AG, our results are, to the best of our knowledge, the first published using twin data.

### Familial aggregation and co-aggregation of diseases

With considerable genetic influence on most AIDs, our findings of higher HRs for (same) disease aggregation in MZ compared to DZ twins across all AIDs was expected. HRs are dependent on disease prevalence, as evident from AD, a rare disorder with a high (MZ) probandwise concordance, yielding an extremely high HR. Comparing HRs for diseases with different prevalence is therefore of limited interest, rather, HRs can be seen as reference points when assessing HRs for co-aggregation. For most AIDs, co-aggregation was lower in DZ than in MZ twins, confirming that genetic components are indeed important in disease clustering. However, in CD, VI, and AD, co-aggregation was equally common in DZ twins. This could be interpreted as predominantly environmental influence on co-aggregation and mostly disease-specific genetic risk contributing to these disorders, but it is also consistent with the ‘common variant/multiple disease’ hypothesis. This states that common alleles predisposing to a given disorder in a particular genetic/environmental setting may contribute to alternate disorders in a different genetic/environmental context (28). Moreover, the high heritability of ‘any disease’, the aggregate of all seven diseases, points to considerable genetic homogeneity among these AIDs. Clustering of all AIDs into one category provided an overview of the autoimmune risk that allowed for direct comparison across index diseases. Overall, AG and GD stood out as the disorders with the lowest risk of autoimmunity in a broader sense, with DZ twins at a modestly increased risk of AID (HRs 1.5 and 1.6). At the other end of the spectrum, AD with a HR of 25.7 (95% CI: 8.8–74.7) in MZ twins and 10.1 (95% CI: 2.5–41.7) in DZ twins stood out as a highly autoimmune condition.

### Heritability

All estimates of heritability presented in this study are based on the AE model, where additive genetic effects (A) and unique environment not shared between twins (E) explain the observed variance. Thus, variance not explained by heritability is attributed to E.

In thyroid autoimmunity, Danish twin cohorts have been used to calculate the heritability of GD (0.79) (15) and of autoantibodies directed against thyroperoxidase and thyroglobulin in euthyroid subjects (0.73) (14). For overt HT, heritability estimates are, to the best of our knowledge, still lacking, but concordance data on twins point to strong genetic influences (29). Our results were in line with previous studies, but returned slightly lower point-estimates and considerably tighter CIs, due to the larger sample sizes. CD has been the focus of repeated twin studies using different cohorts, including the STR. Estimates of heritability range from 0.57 to 0.87 (17, 30), which is somewhat lower than our results of 0.91. A previous study using data from the STR reported a heritability of 0.75 for CD (17), with most of the difference from our estimate explained by a component of shared environmental influence (C) not found in this study. DM is also well studied in this context, albeit in smaller twin cohorts. Finnish and Danish data point to a heritability of 0.72–0.88 (16, 31), well in line with our findings of 0.81. The heritability for AD has recently been charted using data from the STR (18). This study, using a marginally larger cohort but improved statistics, yielded nearly identical results, confirming very high heritability. Reports on twin concordance in VI indicate that genetic effects are present (32), but heritability has not been examined using twins. Family-based studies report a heritability of 0.45–0.72, with most estimates gravitating toward the lower end of that interval (33). Our result of 0.65 suggests that genetics may be of greater importance than previously believed, at least in Caucasian populations. The genetic influence on AG is poorly studied, and heritability estimates are absent. This is probably, in some part, due to the heterogenic etiology of the disease. Both Helicobacter Pylori infections and autoimmunity, sometimes overlapping, can lead to AG (34). In pernicious anemia, a late complication of primarily autoimmune AG studies on family clustering and case reports on twin concordance suggest that genetic factors are important (35). In our study, heritability for AG was considerably lower than for the other examined conditions. This may reflect a non-autoimmune etiology to many of the cases included, but a strong environmental influence (as opposed to a strong heritability) is perhaps not a surprising find, given that H. Pylori can trigger autoimmune AG.

### Strengths and limitations

A major strength of this work is the combined use of an unbiased cohort representing a large majority of all Swedish twins and national health registries with excellent coverage. Using twin data to analyze etiologic overlap for multiple rather than single traits is commonplace in behavioral sciences and psychiatry (36). To the best of our knowledge, this is the first study to apply these methods on autoimmunity. Another strength is that we, unlike previous studies on heritability for AIDs, have used a statistical approach that takes time of follow-up and age into account. This is crucial for relevant heritability estimates, as autoimmunity often presents late in life. These factors, in combination with a high prevalence for AIDs in the population, have enabled us to study AIDs with higher precision than before, as reflected in the tighter CIs for the heritability estimates. A limitation of this study is the use of registry-based data rather than clinical records to ascertain cases. Diagnostic accuracy is generally very good for the NPR (21) and, when possible, we have used prescription data from the PDR to improve accuracy. For DM, AD, HT, GD, and CD, diagnostic precision is probably high and prevalence and sex distribution are in line with population estimates based on biochemical and/or clinical records. For VI, under-reporting is most likely substantial. As previously elaborated, diagnosing autoimmune AG entails additional challenges. Access to clinical records would not necessarily solve this issue, as diagnoses of AG seldomly rely on gastric mucosal biopsies, and serology for AG is uncommon in clinical practice. If MZ twins were screened for diagnoses present in their co-twins more often than DZ twins, this could have distorted results. An indirect sign of surveillance bias would be a higher autoimmune prevalence in MZ than in DZ twins. In this study, AIDs were, in fact, marginally more common in DZ than in MZ twins (Table 1), making this bias unlikely. The AIDs studied here include both common and rare organ-specific disorders. Extrapolating from data on autoimmunity in the Danish population, they account for approximately 55% of the total autoimmune burden (37). Many AIDs not included in our study are known to co-aggregate, and partitioning of AIDs into organ-specific vs systemic diseases is not mirrored in phenotypic presentations. Systemic AIDs sometimes congregate with organ-specific AIDs, including many of the diseases we have explored (8, 38). Consequently, the HRs presented here, and the conclusions drawn based on them, do not necessarily hold true in a different autoimmune panorama.

To conclude, in this study we pioneer the use of twins in examining autoimmune co-aggregation in seven autoimmune conditions of varying prevalence. We also present updated heritability estimates that corroborate and, in some cases, correct previous estimates based on smaller twin cohorts and older statistical methods. Not least because genome-wide association studies have explained only a minority of autoimmune morbidity, and will never explain the discordance in MZ twins, further epidemiological studies are warranted to partition genetic and environmental risk factors as either disease specific or as shared between diseases. Comprehensive information on familial relations in registries covering the whole population would enable detailed dissection of co-inheritance and shared environment, including bivariate analyses across a wide spectrum of diseases.

## Supplementary Material

APPENDIX Co-aggregation and heritability in seven organ-specific autoimmune diseases: a population-based twin study.

supplementary Figure 1.

Supplemental table 1 Heritability. All estimates and Akaikes Information Criterion Adjusted models (sex and birth year), accounting for left-truncation and right-censoring.

## Declaration of interest

The authors declare that there is no conflict of interest that could be perceived as prejudicing the impartiality of this study.

## Funding

The present study was supported by the County Council of Värmland (to J S), County Council of Stockholm (to S B), Swedish Society for Medical Research (to S B), Åke Wiberg Foundation (to S B), Swedish Research Council (to O K), and Novo Nordisk Foundation (to O K). 
