# Supplementary material for: Co-aggregation and heritability of organ-specific autoimmunity: a population-based twin study
Source: Eur J Endocrinol. 2020 Mar 4;182(5):473–80. doi: 10.1530/EJE-20-0049 (PMC7182094; doi:10.1530/EJE-20-0049)
Supplement: APPENDIX Co-aggregation and heritability in seven organ-specific autoimmune diseases: a population-based twin study. [file supplementary_material.pdf]

APPENDIX
Co-aggregation and heritability in seven organ-specific autoimmune diseases: a population-based twin study.

Inclusion and exclusion criteria for Addison's disease

Inclusion criteria
ICD-10 E27.1, E27.2
ICD-9 2554
ICD-8 255,10
ICD-7 274.40

AND

Multiple dispensations of hydrocortisone/cortisone acetate ATC H02AB09/H02AB10 in SPDR in combination with multiple dispensations of fludrocortisone ATC H02AA02.

Exclusion criteria
ICD-10 A15-A19, P37.0, J65, B90, A39.1, B20-B24, C74, C79.7, D35.0, D44.1, E25, E27.3, E27.4, E35.1, E71.3, E89.6, P54.4, Q89.1, E220, E222, E228, E229, E23*, E24*, C75.1, D35.2, D44.3, E89.3
ICD-9 010-018, 137, 0363, 0798, 2799, 1940, 1987, 2270, 2372, 2552, 2555, 3300, 7725, 7591, 253*, 2550, 1943 2273, 2370, 2537
ICD-8 010-019, Y34.09, Y34.19, Y34.29, 036.11, 194.01, 226.00-09, 239.10, 255.01, 255.02, 333.10, 758.10, 253*, 253*, 258.00, 194.31, 226.20, 239.90
ICD-7 001.99-019.20, Y03.00, Y03.10, Y03.20, Y53.00, 057.11, 195.01, 224.10-224.19, 195.00, 239.31, 272*, 277.10, 195.31, 195.30, 224.40, 239.34
* all codes

Inclusion and exclusion criteria for type 1 diabetes
Inclusion criteria:
Patients identified through linkage of the National Diabetes Register (including SWEDIABKIDS) and the Swedish Twin Register.
The Swedish National Diabetes Register was designed for measuring the quality and nationwide equality of diabetes care in Sweden, but it is also widely used in research. Virtually all Swedish patients with diabetes (type-1, type-2, MODY) are included. Type of diabetes is one of the variables entered by the treating physician. On suspicion of type-1 diabetes, autoantibodies and C-peptide levels are routinely checked to confirm diagnosis.

(Time of diagnosis: First record of diabetes, either in the National Diabetes Register or the National Patient Register)

Exclusion criteria:
None

Inclusion and exclusion criteria for Graves' disease
Inclusion criteria:
ICD-10 E050 E055
ICD-9 242A
ICD-8 242,00 242,09
ICD-7 252,00 252,01 252,02

Exclusion criteria:

ICD-10 E051 E052 E053 E054 E058 E060 E061
ICD-9 242B 242C 242E 242W 245A 245B
ICD-8 242,10 245,00 245,01 245,02
ICD-7 252,10 254,10 254,20

Inclusion and exclusion criteria for Hashimoto’s thyroiditis

Inclusion criteria:
ICD-10 E035 E039 E063 E065
ICD-9 244X 245C 245W
ICD-8 244,09 245,03
ICD-7 253,00 253,19 253,29 254,00

Exclusion criteria:
ICD-10 E030 E031 E032 E033 E064 E890 E00 E01 E02 E060 E061
ICD-9 246B 243 244A 244B 244C 244D 244W
ICD-8 243,99 245,00 245,01 245,02 245,04 244,00
ICD-7 250,00 253,10 253,20 254,10 254,20
Or procedural codes
BAA60 BAA40 BAA50 BAA20
810 811 814 820 812 813 822 824 830

Or:

If alive 1 jan 2006; failure to fill at least two prescription for levothyroxine (ATC- H03AA)

Or:

Previous or concurrent Graves’ disease

Alternative inclusion:
A diagnosis of Addison’s disease or type 1 diabetes and at least two filled prescriptions for levothyroxine (ATC-H03AA)

Exclusion from alternative inclusion:

Exklusion diagnosis for Hashimoto’s thyroiditis. Diagnosis or exclusion diagnosis for Graves’ disease.
ICD-10 E059
ICD-9 242X
ICD-8 242,20

Inclusion and exclusion criteria for pernicious anemia (with B12 deficiency) and/or chronic atrophic gastritis

Inclusion criteria:
ICD-10 D510 K293 K294 K295
ICD-9 281A 535B
ICD-8 281,00 281,09 535,03
ICD-7 290,00 290,10 543,01

Exclusion criteria:
Celiac disease*

ICD-10 K900
ICD-9 579A
ICD-8 269,00 269,98
ICD-7 286,00

*In patients with Celiac disease, malabsorption due to incomplete adherence to gluten-free diet was considered equally if not more likely to explain B-12 deficiency than autoimmune atrophic gastritis.

Inclusion and exclusion criteria for celiac disease

Inclusion criteria:
ICD-10 K900
ICD-9 579A
ICD-8 269,00 269,98
ICD-7 286,00

Exclusion criteria:
Lack of ICD-9 or ICD-10 diagnosis for celiac disease if alive after 1997.

Inclusion and exclusion criteria for vitiligo

Inclusion criteria:
ICD-10 L80
ICD-9 709A
ICD-8 709,05
ICD-7 716,11

Exclusion criteria:
None
