# Supplementary material for: Co-aggregation and heritability of organ-specific autoimmunity: a population-based twin study
Source: Eur J Endocrinol. 2020 Mar 4;182(5):473–80. doi: 10.1530/EJE-20-0049 (PMC7182094; doi:10.1530/EJE-20-0049)
Supplement: supplementary Figure 1. [file supplementary_figure_1.pdf]

	HT	AG	CD	GD	DM	VI
AG	11 / 11					
CD	7 / 12	1 / 0				
GD	15 / 16	3 / 3	2 / 6			
DM	11 / 5	0 / 2	2 / 8	1 / 6		
VI	7 / 4	1 / 0	0 / 0	1 / 1	1 / 3	
AD	6 / 1	1 / 0	0 / 0	0 / 2	0 / 1	2 / 0

Supplemental figure 1

Pairwise combinations of autoimmune diseases. The numbers in each box represent twin pairs with one disease present in one twin and the other disease present in the co-twin. MZ twins bottom left, DZ twins top right. The collected data served as substrate for estimation of coaggregations. HT - Hashimoto's thyroiditis, AG - atrophic gastritis, CD -celiac disease, GD - Graves' disease, DM - Type 1 diabetes, VI - vitiligo, AD - Addison's disease.
