# Supplementary material for: Co-aggregation and heritability of organ-specific autoimmunity: a population-based twin study
Source: Eur J Endocrinol. 2020 Mar 4;182(5):473–80. doi: 10.1530/EJE-20-0049 (PMC7182094; doi:10.1530/EJE-20-0049)
Supplement: Supplemental table 1 Heritability. All estimates and Akaikes Information Criterion Adjusted models (sex and birth year), accounting for left-truncation and right-censoring. [file supplementary_table_1.pdf]

Supplemental table 1a

Heritability. All estimates and Akaikes Information Criterion

Adjusted models (sex and birth year), accounting for left-truncation and right-censoring.

	A	D	C	E	A+D	AIC
Hashimoto's disease ACE	0.636 (0.575-0.697)	-	0.000 (-0.000-0.000)	0.364 (0.303-0.425)	0.636 (0.575-0.697)	3164.96
Hashimoto's disease ADE	0.633 (0.405-0.861)	0.003 (-0.235-0.242)	-	0.364 (0.301-0.427)	0.636 (0.573-0.699)	3164.96
Hashimoto's disease AE	0.636 (0.575-0.697)	-	-	0.364 (0.303-0.425)	0.636 (0.575-0.697)	3162.96
Atrophic gastritis ACE	0.378 (0.226-0.530)	-	0.000 (-0.000-0.000)	0.622 (0.470-0.774)	0.378 (0.226-0.530)	1769.92
Atrophic gastritis ADE	0.111 (-0.528-0.751)	0.300 (-0.399-1.000)	-	0.588 (0.424-0.752)	0.412 (0.248-0.576)	1769.71
Atrophic gastritis AE	0.378 (0.226-0.530)	-	-	0.622 (0.470-0.774)	0.378 (0.226-0.530)	1767.92
Celiac disease ACE	0.744 (0.566-0.921)	-	0.155 (-0.009-0.319)	0.101 (0.062-0.140)	0.744 (0.566-0.921)	1205.24
Celiac disease ADE	0.906 (0.871-0.940)	0.000 (-0.000-0.000)	-	0.094 (0.060-0.129)	0.906 (0.871-0.940)	1205.78
Celiac disease AE	0.906 (0.871-0.940)	-	-	0.094 (0.060-0.129)	0.906 (0.871-0.940)	1203.78
Graves' disease ACE	0.600 (0.487-0.713)	-	0.000 (0.000-0.000)	0.400 (0.287-0.513)	0.600 (0.487-0.713)	1980.26
Graves' disease ADE	0.000 (-0.000-0.000)	0.641 (0.534-0.747)	-	0.359 (0.253-0.466)	0.641 (0.534-0.747)	1978.16
Graves' disease AE	0.600 (0.487-0.713)	-	-	0.400 (0.287-0.513)	0.600 (0.487-0.713)	1978.26
Type 1 diabetes ACE	0.805 (0.726-0.885)	-	0.000 (-0.000-0.000)	0.195 (0.115-0.274)	0.805 (0.726-0.885)	3401.59
Type 1 diabetes ADE	0.441 (-0.177-1.059)	0.377 (-0.254-1.009)	-	0.182 (0.106-0.257)	0.818 (0.743-0.894)	3400.37
Type 1 diabetes AE	0.805 (0.726-0.885)	-	-	0.195 (0.115-0.274)	0.805 (0.726-0.885)	3399.59
Vitiligo ACE	0.653 (0.496-0.810)	-	0.000 (0.000-0.000)	0.347 (0.190-0.504)	0.653 (0.496-0.810)	805.57
Vitiligo ADE	0.000 (0.000-0.000)	0.672 (0.521-0.823)	-	0.328 (0.177-0.479)	0.672 (0.521-0.823)	805.01
Vitiligo AE	0.653 (0.496-0.810)	-	-	0.347 (0.190-0.504)	0.653 (0.496-0.810)	803.57
Addison's disease ACE	0.969 (0.914-1.025)	-	0.000 (0.000-0.000)	0.031 (-0.025-0.086)	0.969 (0.914-1.025)	130.61
Addison's disease ADE	0.000 (-0.000-0.000)	0.970 (0.914-1.025)	-	0.030 (-0.025-0.085)	0.970 (0.915-1.025)	130.54
Addison's disease AE	0.969 (0.914-1.025)	-	-	0.031 (-0.025-0.086)	0.969 (0.914-1.025)	128.61
Any disease ACE	0.687 (0.647-0.727)	-	0.000 (-0.000-0.000)	0.313 (0.273-0.353)	0.687 (0.647-0.727)	16054.73
Any disease ADE	0.543 (0.329-0.757)	0.155 (-0.070-0.381)	-	0.301 (0.260-0.342)	0.699 (0.658-0.740)	16053.30
Any disease AE	0.687 (0.647-0.727)	-	-	0.313 (0.273-0.353)	0.687 (0.647-0.727)	16052.73

Supplemental table 1b
Heritability. All estimates and Akaikes Information Criterion
Unadjusted models, accounting for left-truncation and right-censoring.

	A	D	C	E	A+D	AIC
Hashimoto's disease ACE	0.643 (0.472-0.814)	-	0.050 (-0.093-0.192)	0.307 (0.252-0.362)	0.643 (0.472-0.814)	3519.81
Hashimoto's disease ADE	0.700 (0.650-0.749)	0.000 (-0.000-0.000)	-	0.300 (0.251-0.350)	0.700 (0.650-0.749)	3519.91
Hashimoto's disease AE	0.700 (0.650-0.749)	-	-	0.300 (0.251-0.350)	0.700 (0.650-0.749)	3517.91
Atrophic gastritis ACE	0.506 (0.375-0.636)	-	0.000 (-0.000-0.000)	0.494 (0.364-0.625)	0.506 (0.375-0.636)	1972.25
Atrophic gastritis ADE	0.471 (-0.074-1.017)	0.038 (-0.561-0.637)	-	0.491 (0.344-0.638)	0.509 (0.362-0.656)	1972.25
Atrophic gastritis AE	0.506 (0.375-0.636)	-	-	0.494 (0.364-0.625)	0.506 (0.375-0.636)	1970.25
Celiac disease ACE	0.732 (0.560-0.904)	-	0.172 (0.012-0.333)	0.096 (0.059-0.132)	0.732 (0.560-0.904)	1216.15
Celiac disease ADE	0.912 (0.879-0.944)	0.000 (-0.000-0.000)	-	0.088 (0.056-0.121)	0.912 (0.879-0.944)	1216.86
Celiac disease AE	0.912 (0.879-0.944)	-	-	0.088 (0.056-0.121)	0.912 (0.879-0.944)	1214.86
Graves' disease ACE	0.652 (0.552-0.751)	-	0.000 (0.000-0.000)	0.348 (0.249-0.448)	0.652 (0.552-0.751)	2171.61
Graves' disease ADE	0.000 (-0.000-0.000)	0.684 (0.591-0.777)	-	0.316 (0.223-0.409)	0.684 (0.591-0.777)	2170.08
Graves' disease AE	0.652 (0.552-0.751)	-	-	0.348 (0.249-0.448)	0.652 (0.552-0.751)	2169.61
Type 1 diabetes ACE	0.819 (0.746-0.893)	-	0.000 (-0.000-0.000)	0.181 (0.107-0.254)	0.819 (0.746-0.893)	3532.48
Type 1 diabetes ADE	0.577 (-0.027-1.182)	0.250 (-0.367-0.867)	-	0.173 (0.101-0.245)	0.827 (0.755-0.899)	3531.92
Type 1 diabetes AE	0.819 (0.746-0.893)	-	-	0.181 (0.107-0.254)	0.819 (0.746-0.893)	3530.48
Vitiligo ACE	0.657 (0.501-0.812)	-	0.000 (0.000-0.000)	0.343 (0.188-0.499)	0.657 (0.501-0.812)	793.40
Vitiligo ADE	0.000 (0.000-0.000)	0.675 (0.526-0.824)	-	0.325 (0.176-0.474)	0.675 (0.526-0.824)	792.85
Vitiligo AE	0.657 (0.501-0.812)	-	-	0.343 (0.188-0.499)	0.657 (0.501-0.812)	791.40
Addison's disease ACE	0.973 (0.923-1.022)	-	0.000 (-0.000-0.000)	0.027 (-0.022-0.077)	0.973 (0.923-1.022)	117.79
Addison's disease ADE	0.000 (-0.000-0.000)	0.973 (0.924-1.022)	-	0.027 (-0.022-0.076)	0.973 (0.924-1.022)	117.71
Addison's disease AE	0.973 (0.923-1.022)	-	-	0.027 (-0.022-0.077)	0.973 (0.923-1.022)	115.79
Any disease ACE	0.708 (0.672-0.744)	-	0.000 (-0.000-0.000)	0.292 (0.256-0.328)	0.708 (0.672-0.744)	16822.72
Any disease ADE	0.577 (0.381-0.772)	0.141 (-0.064-0.346)	-	0.282 (0.245-0.319)	0.718 (0.681-0.755)	16821.49
Any disease AE	0.708 (0.672-0.744)	-	-	0.292 (0.256-0.328)	0.708 (0.672-0.744)	16820.72

Supplemental table 1c
Heritability. All estimates and Akaikes Information Criterion
Adjusted models (sex and birth year), not accounting for left-truncation and right-censoring.

	A	D	C	E	A+D	AIC
Hashimoto's disease ACE	0.617 (0.433-0.801)	-	0.023 (-0.128-0.174)	0.360 (0.299-0.421)	0.617 (0.433-0.801)	15267.14
Hashimoto's disease ADE	0.644 (0.589-0.699)	0.000 (-0.000-0.000)	-	0.356 (0.301-0.411)	0.644 (0.589-0.699)	15267.24
Hashimoto's disease AE	0.644 (0.589-0.699)	-	-	0.356 (0.301-0.411)	0.644 (0.589-0.699)	15265.24
Atrophic gastritis ACE	0.380 (0.250-0.510)	-	0.000 (-0.000-0.000)	0.620 (0.490-0.750)	0.380 (0.250-0.510)	7309.06
Atrophic gastritis ADE	0.245 (-0.309-0.798)	0.154 (-0.452-0.759)	-	0.602 (0.458-0.746)	0.398 (0.254-0.542)	7308.83
Atrophic gastritis AE	0.380 (0.250-0.510)	-	-	0.620 (0.490-0.750)	0.380 (0.250-0.510)	7307.06
Celiac disease ACE	0.750 (0.576-0.924)	-	0.148 (-0.013-0.309)	0.102 (0.064-0.140)	0.750 (0.576-0.924)	7226.47
Celiac disease ADE	0.904 (0.870-0.938)	0.000 (-0.000-0.000)	-	0.096 (0.062-0.130)	0.904 (0.870-0.938)	7229.33
Celiac disease AE	0.904 (0.870-0.938)	-	-	0.096 (0.062-0.130)	0.904 (0.870-0.938)	7227.33
Graves' disease ACE	0.594 (0.485-0.702)	-	0.000 (0.000-0.000)	0.406 (0.298-0.515)	0.594 (0.485-0.702)	6381.81
Graves' disease ADE	0.000 (-0.000-0.000)	0.633 (0.530-0.736)	-	0.367 (0.264-0.470)	0.633 (0.530-0.736)	6375.94
Graves' disease AE	0.594 (0.485-0.702)	-	-	0.406 (0.298-0.515)	0.594 (0.485-0.702)	6379.81
Type 1 diabetes ACE	0.818 (0.745-0.892)	-	0.000 (-0.000-0.000)	0.182 (0.108-0.255)	0.818 (0.745-0.892)	4496.21
Type 1 diabetes ADE	0.597 (0.003-1.192)	0.229 (-0.378-0.836)	-	0.174 (0.102-0.246)	0.826 (0.754-0.898)	4495.63
Type 1 diabetes AE	0.818 (0.745-0.892)	-	-	0.182 (0.108-0.255)	0.818 (0.745-0.892)	4494.21
Vitiligo ACE	0.646 (0.490-0.801)	-	0.000 (0.000-0.000)	0.354 (0.199-0.510)	0.646 (0.490-0.801)	2508.17
Vitiligo ADE	0.000 (0.000-0.000)	0.664 (0.515-0.814)	-	0.336 (0.186-0.485)	0.664 (0.515-0.814)	2506.53
Vitiligo AE	0.646 (0.490-0.801)	-	-	0.354 (0.199-0.510)	0.646 (0.490-0.801)	2506.17
Addison's disease ACE	0.970 (0.914-1.025)	-	0.000 (-0.000-0.000)	0.030 (-0.025-0.086)	0.970 (0.914-1.025)	442.61
Addison's disease ADE	0.000 (NaN-NaN)	0.970 (0.916-1.024)	-	0.030 (-0.024-0.084)	0.970 (0.916-1.024)	442.32
Addison's disease AE	0.970 (0.914-1.025)	-	-	0.030 (-0.025-0.086)	0.970 (0.914-1.025)	440.61
Any disease ACE	0.635 (0.598-0.673)	-	0.000 (-0.000-0.000)	0.365 (0.327-0.402)	0.635 (0.598-0.673)	31523.67
Any disease ADE	0.486 (0.301-0.671)	0.164 (-0.034-0.362)	-	0.350 (0.310-0.390)	0.650 (0.610-0.690)	31520.83
Any disease AE	0.635 (0.598-0.673)	-	-	0.365 (0.327-0.402)	0.635 (0.598-0.673)	31521.67

Supplemental table 1d
Heritability. All estimates and Akaikes Information Criterion
Unadjusted models, not accounting for left-truncation and right-censoring.

	A	D	C	E	A+D	AIC
Hashimoto’s disease ACE	0.615 (0.456-0.775)	-	0.081 (-0.051-0.214)	0.303 (0.252-0.355)	0.615 (0.456-0.775)	16939.90
Hashimoto’s disease ADE	0.708 (0.662-0.754)	0.000 (-0.000-0.000)	-	0.292 (0.246-0.338)	0.708 (0.662-0.754)	16941.32
Hashimoto’s disease AE	0.708 (0.662-0.754)	-	-	0.292 (0.246-0.338)	0.708 (0.662-0.754)	16939.32
Atrophic gastritis ACE	0.456 (0.085-0.827)	-	0.017 (-0.272-0.305)	0.527 (0.393-0.662)	0.456 (0.085-0.827)	7956.39
Atrophic gastritis ADE	0.476 (0.358-0.595)	0.000 (-0.000-0.000)	-	0.524 (0.405-0.642)	0.476 (0.358-0.595)	7956.40
Atrophic gastritis AE	0.476 (0.358-0.595)	-	-	0.524 (0.405-0.642)	0.476 (0.358-0.595)	7954.40
Celiac disease ACE	0.725 (0.557-0.893)	-	0.179 (0.022-0.335)	0.096 (0.060-0.132)	0.725 (0.557-0.893)	7433.44
Celiac disease ADE	0.911 (0.879-0.943)	0.000 (-0.000-0.000)	-	0.089 (0.057-0.121)	0.911 (0.879-0.943)	7438.01
Celiac disease AE	0.911 (0.879-0.943)	-	-	0.089 (0.057-0.121)	0.911 (0.879-0.943)	7436.01
Graves’ disease ACE	0.641 (0.544-0.738)	-	0.000 (0.000-0.000)	0.359 (0.262-0.456)	0.641 (0.544-0.738)	6913.90
Graves’ disease ADE	0.000 (-0.000-0.000)	0.674 (0.582-0.765)	-	0.326 (0.235-0.418)	0.674 (0.582-0.765)	6909.31
Graves’ disease AE	0.641 (0.544-0.738)	-	-	0.359 (0.262-0.456)	0.641 (0.544-0.738)	6911.90
Type 1 diabetes ACE	0.831 (0.763-0.899)	-	0.000 (-0.000-0.000)	0.169 (0.101-0.237)	0.831 (0.763-0.899)	4700.57
Type 1 diabetes ADE	0.717 (0.143-1.291)	0.117 (-0.468-0.703)	-	0.166 (0.097-0.234)	0.834 (0.766-0.903)	4700.42
Type 1 diabetes AE	0.831 (0.763-0.899)	-	-	0.169 (0.101-0.237)	0.831 (0.763-0.899)	4698.57
Vitiligo ACE	0.654 (0.502-0.806)	-	0.000 (0.000-0.000)	0.346 (0.194-0.498)	0.654 (0.502-0.806)	2531.72
Vitiligo ADE	0.000 (0.000-0.000)	0.671 (0.524-0.818)	-	0.329 (0.182-0.476)	0.671 (0.524-0.818)	2530.16
Vitiligo AE	0.654 (0.502-0.806)	-	-	0.346 (0.194-0.498)	0.654 (0.502-0.806)	2529.72
Addison’s disease ACE	0.973 (0.923-1.022)	-	0.000 (NaN-NaN)	0.027 (-0.022-0.077)	0.973 (0.923-1.022)	449.96
Addison’s disease ADE	0.000 (NaN-NaN)	0.973 (0.924-1.022)	-	0.027 (-0.022-0.076)	0.973 (0.924-1.022)	449.69
Addison’s disease AE	0.973 (0.923-1.022)	-	-	0.027 (-0.022-0.077)	0.973 (0.923-1.022)	447.96
Any disease ACE	0.659 (0.624-0.695)	-	0.000 (-0.000-0.000)	0.341 (0.305-0.376)	0.659 (0.624-0.695)	32795.00
Any disease ADE	0.510 (0.334-0.685)	0.163 (-0.024-0.350)	-	0.327 (0.290-0.364)	0.673 (0.636-0.710)	32792.04
Any disease AE	0.659 (0.624-0.695)	-	-	0.341 (0.305-0.376)	0.659 (0.624-0.695)	32793.00
